# Using evolutionary context to classify difficult protein folds

**DOI:** 10.1371/journal.pcbi.1013773

**Published:** 2025-12-01

**Authors:** Jimin Pei, R. Dustin Schaeffer, Qian Cong, Nick V. Grishin

**Affiliations:** 1 Eugene McDermott Center for Human Growth and Development, University of Texas Southwestern Medical Center, Dallas, Texas, United States of America; 2 Department of Biophysics, University of Texas Southwestern Medical Center, Dallas, Texas, United States of America; 3 Harold C. Simmons Comprehensive Cancer Center, University of Texas Southwestern Medical Center, Dallas, Texas, United States of America; 4 Department of Biochemistry, University of Texas Southwestern Medical Center, Dallas, Texas, United States of America; Seoul National University, KOREA, REPUBLIC OF

## Abstract

Recent advances in protein structure prediction, such as AlphaFold2, have enabled identification of vast numbers of putative novel protein domains across the sequence space, many of which adopt structures dissimilar to known folds. Based on structural segmentation and classification, the Encyclopedia of Domains (TED) project recently cataloged more than 7400 low-symmetry, structure-based domains as candidate novel-fold (CNF) domains. To place these domains in their broader evolutionary and structural context, we applied DPAM (Domain Parser for AlphaFold Models), a complementary method that combines AlphaFold-derived confidence metrics with sensitive sequence and structure similarity searches, to parse domains for the AlphaFold models of proteins containing TED CNF domains. We identified 8044 DPAM domains with significant overlap with TED CNF domains, among which 2490 were confidently assigned to entries in the ECOD (Evolutionary Classification of protein Domains) structural classification hierarchy. Our results suggest that a substantial subset of TED candidate novel-fold domains are distant homologs of existing ECOD domains. Comparison of domain boundaries between TED and DPAM showed varied patterns: more than one-third of cases featured TED CNF domains largely embedded within DPAM domains—often representing insertions or extensions into enzymatic or repeat folds. A smaller fraction (17%) exhibited consistent domain boundaries between TED and DPAM. These consistently defined domains are often characterized by significant structural diversity, including long insertions and duplications. An even smaller subset showed the reverse relationship, with DPAM domains largely embedded within TED CNF domains. In these cases, DPAM effectively separated multiple structural units that TED grouped as single domains. Together, these findings highlight the complementarity of structural and evolutionary approaches for domain annotation and demonstrate the power of integrative methods, such as DPAM, in refining the classification of challenging protein folds and uncovering distant evolutionary relationships.

## Author summary

In this work, we aimed to understand how protein domains — the fundamental building blocks of proteins — evolve and relate to one another, especially when their shapes are very difficult to classify. Recent advances in protein structure prediction have uncovered thousands of potential “new folds,” making it challenging to place these domains within existing classification systems. To address this, we applied DPAM, which uses information from both protein sequences and predicted structures, to better define and classify these domains. By comparing DPAM results to those from the TED project, we discovered that many TED-defined candidate novel-fold domains correspond to domains inserted into enzymatic or repeat domains and represent highly diverged relatives of known protein families. In other cases, DPAM identified smaller or larger units within TED’s proposed domains, highlighting the complex architecture of many proteins. This work shows how combining structure and sequence information can clarify the evolutionary relationships of challenging protein domains. We believe that this approach will help researchers more accurately annotate protein families, shed light on how new protein folds evolve, and aid in the functional understanding of the growing number of protein structures available from predictive tools.

## Introduction

Protein domains are the fundamental structural and functional units of proteins, often representing distinct evolutionary modules that can exist independently or in combination with other domains [1]. Domains are often associated with specific biological functions, such as enzymatic activity, molecular recognition, or structural stability. Over evolutionary timescales, domains have been recombined, duplicated, and modified, leading to the vast diversity of proteins observed across different organisms [2,3]. Despite the enormous variety in protein sequences, structural studies have shown that proteins tend to adopt a limited number of stable folds, suggesting that structural constraints play a significant role in shaping protein evolution [4]. Some domains are highly conserved across species, while others have diverged significantly, making it challenging to classify them based on sequence similarity alone. The inference of function based on domain homology helps researchers develop biological insights [5,6]. Understanding the evolution and classification of protein domains is crucial for functional annotation, structural modeling, and exploring novel protein engineering applications.

Defining what constitutes a domain remains challenging and can be ambiguous, as different criteria—structural compactness, evolutionary conservation, or functional modularity—can lead to different boundaries (Ponting & Russell, 2002). For example, structural biology often emphasizes compact and spatially distinct three-dimensional units, sequence analysis highlights homologous regions preserved through evolution, and biochemical studies focus on regions with experimentally assigned functions. Over the past few decades, computational programs and resources have been developed to classify protein domains based on sequence and structural similarities, including Pfam [7], CDD [8], SUPERFAMILY [9], SCOP [10], SCOPe [11], CATH [12], and ECOD (Evolutionary Classification of protein Domains) [13]. Different sequence and structural databases of domain classifications apply their own operational rules, which do not always agree [14–17]. This ambiguity means differences in domain boundaries and situations where one framework considers a domain may be treated as several distinct domains in another.

The recent development of highly accurate structure prediction software such as AlphaFold2 [18], RoseTTAFold [19] and ESMFold [20] has led to a wealth of predicted structural data for proteins and protein families that have not yet been structurally characterized by experiment. The release of predicted structures for over 200 million proteins representing the known protein universe by the AlphaFold Structure Database (AFDB) creates an unprecedented breakthrough in predicted protein structural data collection [21]. Classification and analysis of these predicted structures reveal unexplored regions of the protein universe, help define more precise domain boundaries in sequence-based classifications, and provide critical insights into how prediction algorithms perform on proteins evolutionarily distant from those used in training datasets [22–24].

A major issue in protein domain classification is identifying protein domains with no detectable structural similarity to known folds. These domains that adopt potential novel folds present a challenge for classification schemes that rely on structure-based clustering. Even advanced deep learning models, which have dramatically improved structural prediction, are often limited when dealing with completely novel topologies. Without structural homologs to compare against, predicting the function of such proteins becomes particularly difficult, necessitating experimental validation or the development of new computational strategies for detecting subtle sequence-structure relationships.

The TED (The Encyclopedia of Domains) project [24] represents a comprehensive effort to catalog and annotate the full diversity of protein domain architectures across over 200 million predicted structures from AFDB. TED aims to identify, parse, and classify domains across the protein universe, encompassing both well-characterized and previously unannotated domains. TED integrates several recently developed machine learning-based domain parsing methods to carry out domain segmentation [24]. The system leverages fast structural search tools like Foldseek [25] to efficiently assign protein domains to the established CATH classification hierarchy. Remarkably, TED identified 7429 domains with potential novel folds after filtering out domains with pronounced internal repeats or higher-order internal symmetry. The filtering step is vital as high-symmetry domains are often linked to repeat-based or well-characterized folds, and excluding them reduces false positives in novelty detection. As these low-symmetry TED domains show little structural similarity to known structures, we designate them as TED candidate novel-fold (CNF) domains.

To gain deeper insight into the structural and evolutionary context of the challenging TED CNF domains, we applied our recently developed domain parser, DPAM (Domain Parser for AlphaFold Models) [26], to the set of proteins containing these domains. Unlike TED, which relies on structure-based domain parsing, DPAM integrates a broader spectrum of information, including AlphaFold-derived distance maps (distograms), Predicted Aligned Error (PAE) matrices, and sequence profiles. Crucially, DPAM leverages evolutionary context through HHpred searches [27] and enhances structural comparison sensitivity using tools such as DaliLite [28], which are more adept at detecting structural similarities than faster, less sensitive methods like Foldseek. These capabilities allow DPAM to uncover potential evolutionary relationships that may be obscured in TED’s purely structural approach. By assigning the resulting domains to the ECOD structural classification hierarchy, DPAM offers a complementary and potentially more evolutionarily informed perspective, which can classify some TED CNF domains with difficult folds as highly divergent members of known structural lineages. Integrating a method like DPAM into TED could significantly improve the annotation of these domains by providing evolutionary context that structural methods may miss.

## Results and discussion

### Overall statistics of DPAM assignment for TED domains with candidate novel folds

We applied our domain-parsing tool, DPAM, to the AlphaFold models of the 7232 proteins corresponding to the dataset of 7427 TED candidate novel-fold (CNF) domains. A total of 8044 DPAM domains were found to have significant overlap with TED CNF domains (see Materials and methods). The mapping of these DPAM domains to the ECOD database was reported and classified into four categories: well-assigned domains, unassigned domains, partial domains, and simple-topology domains (see Materials and methods). More than half of the DPAM domains (4820 domains, about 60%) were unassigned domains, suggesting a lack of significant evolutionary relationships with ECOD domains. Partial domains (401 domains, about 5%) and simple-topology domains (333 domains, about 4%) have small fractions. Interestingly, nearly one-third of the DPAM domains (2490 domains, about 31%) are well-assigned domains, suggesting that they are evolutionarily related to ECOD domains with experimental structures.

**Fig 1:**
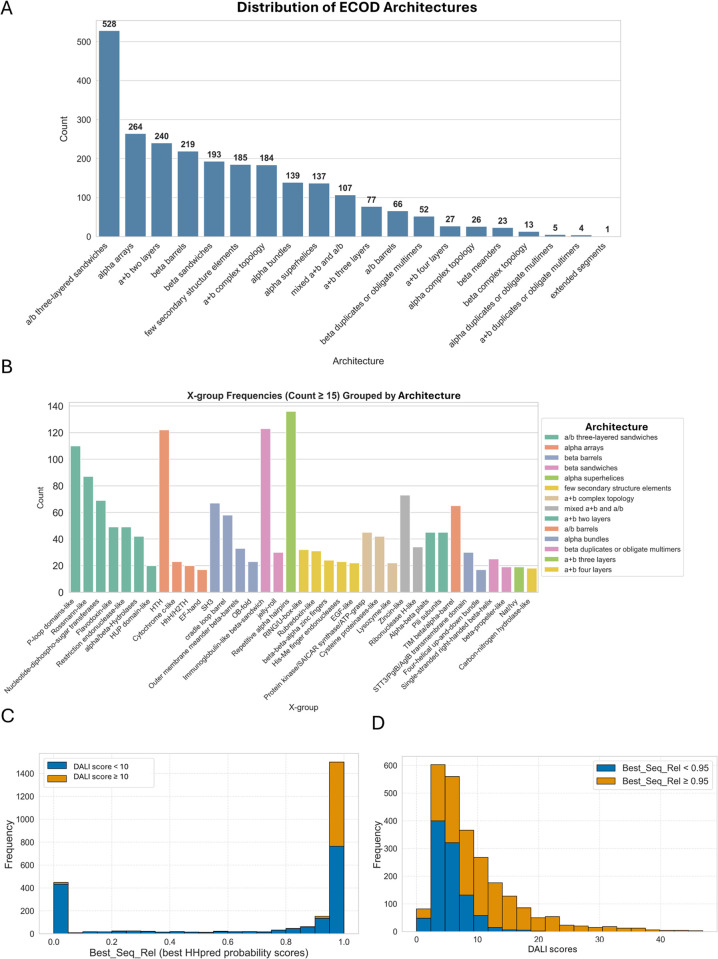
Statistics of ECOD assignments and distributions of sequence and structural similarity scores of well-assigned DPAM domains. **A**. The distribution of ECOD architectures for well-assigned DPAM domains. **B**. The most frequently occurring X-groups in each ECOD architecture. Only X-groups with 15 or more occurrences are shown. **C**. The distribution of Best_Seq_Rel scores of well-assigned DPAM domains. Each bar consists of cases where the DALI score is less than 10 (blue) and cases where the DALI score is 10 or above (orange). **D**. The distribution of Dali scores of well-assigned domains. Each bar consists of cases where the Best_Seq_Rel score is less than 0.95 (blue) and cases where the Best_Seq_Rel score is 0.95 or above (orange).

The 2490 well-assigned DPAM domains represent a broad range of structural and evolutionary characteristics. This set comprises a diverse distribution of protein architectures (ECOD levels indicating overall secondary structure composition and spatial arrangement) and X-groups (ECOD levels indicating possible homology), reflecting a broad range of structural classifications. Among the assigned ECOD architectures, the most prevalent category is “a/b three-layered sandwiches,” comprising approximately 21.2% of the dataset (528 instances). This is followed by “alpha arrays” at 10.6% (264 instances) and “a+b two layers” at 9.6% (240 instances). Other frequently observed architectures include “beta barrels” (8.8%) and “beta sandwiches” (7.8%), highlighting a strong representation of both mixed alpha-beta structures and predominantly β-stranded configurations. On the other hand, other ECOD architectures are less represented, such as “a+b four layers” (27), “alpha complex topology” (26), “beta meanders” (23), and “beta complex topology” (13) (Fig 1A).

The assigned ECOD X-groups exhibit a more fragmented distribution, with no single dominant category (most frequently occurring X-groups in each architecture shown Fig 1B). The most common X-group is “Repetitive alpha hairpins” from the “alpha superhelices” architecture, accounting for 5.5% (136 instances), followed by “Immunoglobulin-like beta-sandwich” of the “beta-sandwiches” architecture at 4.9% (123 instances). Additionally, “HTH” (helix-turn-helix motifs) from the “alpha arrays” architecture represents 4.9% (122 instances). In comparison, “P-loop domains-like” and “Rossmann-like” domains from the “alpha/beta three-layered sandwiches” architecture contribute 4.4% and 3.5% of the dataset, respectively. This variety in X-group classifications underscores the functional and evolutionary diversity captured in the dataset.

To assess sequence-based evolutionary relationships, we define the best sequence similarity metric (Best_Seq_Rel) as the highest HHpred probability score against the ECOD and PDB databases. Domains with a Best_Seq_Rel score above 0.95 are considered to have strong sequence-based evolutionary relationships, while those below 0.8 lack sufficient sequence similarity to known ECOD domains. Based on this classification, 1,503 domains (about 60%, Fig 1C) show significant sequence similarity (Best_Seq_Rel > 0.95), strongly supporting evolutionary relationships based on sequence data. These domains likely belong to well-characterized protein families with clear homologous structures. In contrast, 730 domains (about 29%) have low sequence similarity (Best_Seq_Rel < 0.8), suggesting that their evolutionary relationships cannot be confidently inferred based on sequence alone. 457 domains have a very low Best_Seq_Rel score (below 0.1, Fig 1C). This indicates that a substantial fraction of this dataset of well-assigned domains consists of protein domains whose evolutionary history may be better understood through structural rather than through sequence-based comparisons.

The DALI score (measured by the Dali Z-score of the best ECOD template) provides insight into structural conservation across the dataset. The average DALI score is 9.18, with values ranging from 0 to 46.9 (Fig 1D). The median score is 7.05, indicating that while some domains share strong structural similarity with known templates, many exhibit only moderate resemblance. The standard deviation of 6.99 reflects the wide variability in structural conservation across the dataset and the effect of domain lengths, as shorter domains tend to have lower Dali Z-scores (a structural similarity measure between two structures derived from comparing their intramolecular distance matrices) [29]. Notably, high DALI scores (above 10) suggest close structural homology, even in cases where sequence identity is low, supporting the idea that some domains have diverged significantly at the sequence level while retaining conserved structural folds. About half of the DPAM domains with Best_Seq_Rel scores above 0.95 also achieve a DALI score greater than 10, whereas such cases are rare among domains with Best_Seq_Rel scores below 0.95 (Fig 1C). Structurally, these domains exhibit a moderate level of secondary structure complexity, with around 5 α-helices (mean value: 5.07) and around 6 β-strands (mean value: 5.89) per domain. The low transmembrane segment count (0.05 per domain) suggests that these domains are predominantly soluble, with very few membrane-associated cases.

This dataset captures a rich landscape of protein domain relationships. The strong sequence similarity observed in a majority of cases aligns well with existing classification systems, reinforcing known evolutionary groupings. However, the presence of a significant number of domains with low sequence identity but varying degrees of structural conservation underscores the importance of combining sequence and structural comparisons in understanding protein evolution. The diversity of X-groups in different architectural classifications further highlights the dataset’s broad functional and evolutionary spectrum.

**Fig 2:**
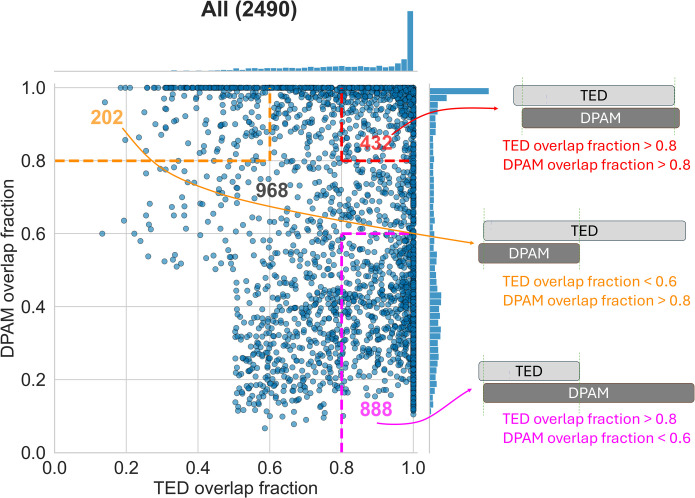
Comparison of overlap fractions of well-assigned DPAM domains and corresponding TED CNF domains. 432 domains are consistently defined domains with both DPAM overlap fraction and TED overlap fraction greater than 0.8 (bounded by red dashed lines). 888 domains have DPAM overlap fraction less than 0.6 and TED overlap fraction greater than 0.8 (bounded by magenta dashed lines), suggesting that TED CNF domains are embedded mainly in the ECOD domains. 202 domains have TED overlap fraction less than 0.6 and DPAM overlap fraction greater than 0.8 (bounded by orange dashed lines), suggesting that ECOD domains are primarily embedded in the TED CNF domains. The remaining 968 domains fall into intermediate cases where neither definition is largely embedded in the other, indicating partial overlaps or more complex boundary relationships between TED and DPAM.

We used overlap fraction to quantify the consistency between DPAM and TED domain definitions (Fig 2). Two overlap fractions are defined: the TED overlap fraction, calculated as the number of residues shared between a DPAM domain and a TED CNF domain divided by the length of the TED CNF domain; and the DPAM overlap fraction, calculated as the number of shared residues divided by the length of the DPAM domain. If both DPAM and TED overlap fractions are high (e.g., above 0.8), DPAM and TED definitions are mostly consistent, a condition met only for 432 DPAM domains (about 17%). Surprisingly, more domains exhibit a high overlap fraction of one definition (above 0.8) and a low overlap fraction of the other definition (below 0.6) (upper left corner or lower right corner in Fig 2 left panel), suggesting that one domain definition is largely embedded within the other domain definition.

### TED CNF domains largely embedded in DPAM domains

More than one third of the well-assigned DPAM domains (888 out of 2490 domains, Fig 2) exhibit a high TED overlap fraction (>0.8) and a low DPAM overlap fraction (<0.6), suggesting that TED CNF domain is considerably shorter than the DPAM domain and is largely embedded in the DPAM domain. This subset of domains spans 18 unique ECOD architectural classifications and 114 distinct X-groups, indicating that they are structurally diverse and occur across a wide range of protein families.

**Fig 3:**
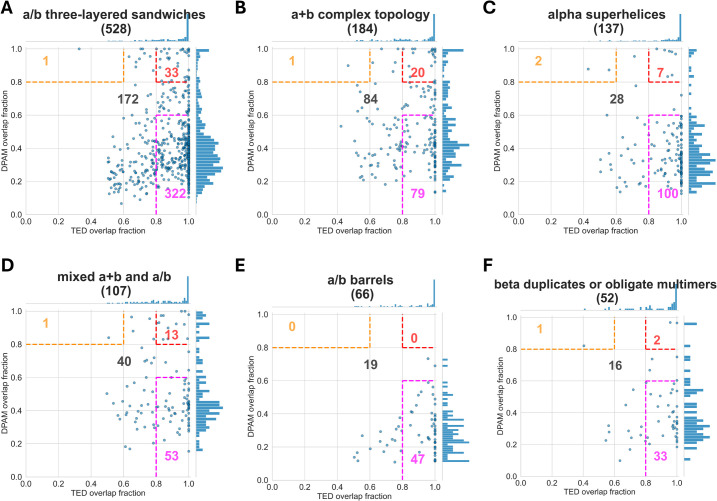
DPAM overlap fractions and TED overlap fractions for ECOD architectures that show predominance of TED CNF domains embedded in DPAM domains. The names of the architectures are shown as the titles of the plots with the number of domains in parentheses. Consistently defined domains are those with DPAM overlap fraction and TED overlap fraction greater than 0.8 (top right corner bounded by red dashed lines, with the number of domains shown in red). TED CNF domains largely embedded in DPAM domains have a DPAM overlap fraction less than 0.6 and a TED overlap fraction greater than 0.8 (bottom right corner bounded by magenta dashed lines, with the number of domains shown in magenta). DPAM domains largely embedded in TED CNF domains have TED overlap fraction less than 0.6 and DPAM overlap fraction greater than 0.8 (top left corner bounded by orange dashed lines, with the number of domains shown in orange). The numbers of domains not in the color-bounded regions are shown in black.

Interestingly, different ECOD architectures exhibit different statistics of the overlap fractions. The cases of TED CNF domains largely embedded in DPAM domains were mostly observed in the architectures of “a/b three-layered sandwiches” (a: alpha; b: beta), “a+b complex topology”, “alpha-superhelices”, “mixed a+b and a/b”, “a/b barrels”, and “beta duplicates or obligate multimers” (Fig 3). For example, among the 528 domains with the “a/b three-layered sandwiches” architecture, more than 50% (322 domains) have TED overlap fraction greater than 0.8 and DPAM overlap fraction less than 0.6, suggesting that the TED CNF domain corresponds to a fraction of the region defined by the DPAM domain. In many cases, TED CNF domain-defined regions are compact and topologically distinct, consistent with the definition of a structural domain, and they may, in principle, fold or function independently. At the same time, some of these regions pack closely against adjacent domains and form extensive interfaces, properties often associated with subdomains rather than fully autonomous domains. Distinguishing between a true domain and a subdomain is not always straightforward, as the criteria for domain definition—structural compactness, evolutionary continuity, or functional modularity—are not fully congruent and can be subjective. Without experimental studies of folding, stability, or functional independence, it is difficult to determine whether these TED CNF domains represent bona fide domains or context-dependent substructures. Thus, while TED highlights them as distinct entities, their classification ultimately reflects the broader ambiguity inherent in defining domain boundaries.

In contrast, only one case shows the opposite domain overlapping scenario (TED overlap fraction < 0.6 and DPAM overlap fraction > 0.8) in the architectures of “a/b three-layered sandwiches” (top left corner in Fig 3A). This trend is seen in all six architectures in Fig 3. The fractions of consistently defined domains (both DPAM and TED overlap fractions >0.8, red-labeled numbers in the top right corners of panels in Fig 3) in these architectures are also low.

**Fig 4:**
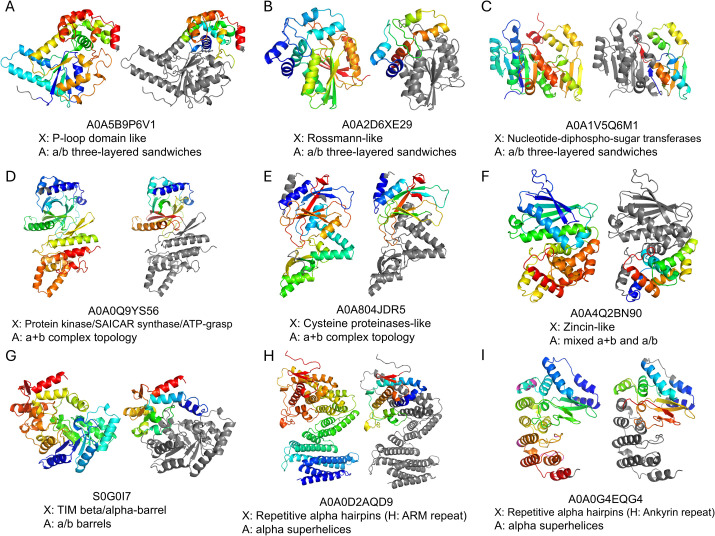
Examples of TED CNF domains largely embedded in DPAM domains. In each example, the right structure depicts the TED CNF domain in rainbow colors, with the remaining regions shown in gray. Note that these gray regions in the right structures may include TED domains that are not classified as CNF domains. The left structure shows the DPAM domain (overlapping with the TED CNF domain) in rainbow colors, with non-DPAM regions shown in gray. The UniProt accession number, the ECOD X-group and the ECOD architecture are shown for each domain. Details on the residue ranges of DPAM domains and TED CNF domains, along with the ECOD assignments and best-matching templates, are available at: http://conglab.swmed.edu/ted_web/dpam90.html.

Analysis of major ECOD X-groups in these architectures suggests that enzyme domains significantly contribute to this category, with TED CNF domains mostly contained within DPAM domains. Examples include X: “P-loop domain like” and X: “Rossmann-like” in the architecture of “a/b three-layered sandwiches”, X: “Protein kinase/SAICAR synthase/ATP-grasp” and X: “Cysteine proteinases-like” in the architecture of “a+b complex topology”, X: “Zincin-like” and X: “Ribonuclease H-like” in the architecture of “mixed a+b and a/b”, and X: “TIM beta/alpha-barrel” in the architecture of “a/b barrels”. Manual inspection of these cases suggests that TED CNF domains often correspond to regions inserted into the core enzyme domains (Fig 4A–4G). While TED defines the CNF domain as an independent domain, DPAM domain definition considers the TED CNF domain and the core enzyme domain together as a single domain. For example, in the protein with UniProt accession S0G0I7, TED defines a CNF domain (TED02: residues 198–234 and 282–371, shown in rainbow on the right structure of Fig 4G) as separate from the TIM-barrel domain (TED01: residues 2–194 and 235–278, shown in the gray region on the right structure of Fig 4G). In contrast, DPAM considers the entire protein (residues 1–370, shown in rainbow on the left structure of Fig 4G) as a single domain assigned to the ECOD X-group of “TIM beta/alpha-barrel”. Other significant X-groups include “Outer membrane meander beta-barrels”, which are commonly found in bacterial outer membrane proteins, suggesting that some insertions/extensions are involved in structural stability or transport functions.

The other two architectures, dominated by TED CNF domains embedded in DPAM domains, are the “alpha superhelices” (Fig 3C) and “beta duplicates and obligate multimers” (Fig 3F), which usually represent repeating structures rather than enzyme domains. For example, out of 137 domains of the “alpha superhelices” architecture, 100 domains have a high TED overlap fraction and a low DPAM fraction, while only two have a low TED fraction and a high DPAM fraction (Fig 3C). This architecture mostly has domains from the X group of “Repetitive alpha hairpins”. Within this X-group, the majority of the domains belong to the H-group of “ARM repeat” (Fig 4H), and the next most frequent H-group is “Ankyrin repeat” (Fig 4I). These α-helical repeats often have insertion regions or extensions in the N- and C-termini serving as cap domains that can function to stabilize the structural repeats [30,31]. These insertion regions and cap domains have been classified together with the α-helical repeat domains as single DPAM domains, while they were considered separate domains by TED definition. Similarly, structures with repeating units were mostly found in the “beta duplicates and obligate multimers” architecture, including X: “Single-stranded right-handed beta-helix” (such as Leucine-rich repeats and Pentapeptide repeats) and X: “beta-propeller-like”, where TED-defined novel fold domains often correspond to their capping motifs [32].

Overall, this subset suggests that many TED CNF domains correspond to regions that DPAM groups into larger composite domains. In many cases, these TED-defined CNF regions could indeed represent compact structural domains, whereas DPAM merges them with surrounding domains. This difference reflects the inherent ambiguity in domain definitions, where structural segmentation and evolutionary classification may produce distinct but complementary boundaries. These TED CNF domains may enhance functional specificity, introduce regulatory elements, or adapt proteins for specialized interactions or environments. The strong sequence conservation of many TED-defined regions suggests that these insertions are not random but rather evolutionarily selected for distinct functional advantages, potentially playing key roles in the expansion and specialization of protein families.

### Consistently defined domains

**Fig 5:**
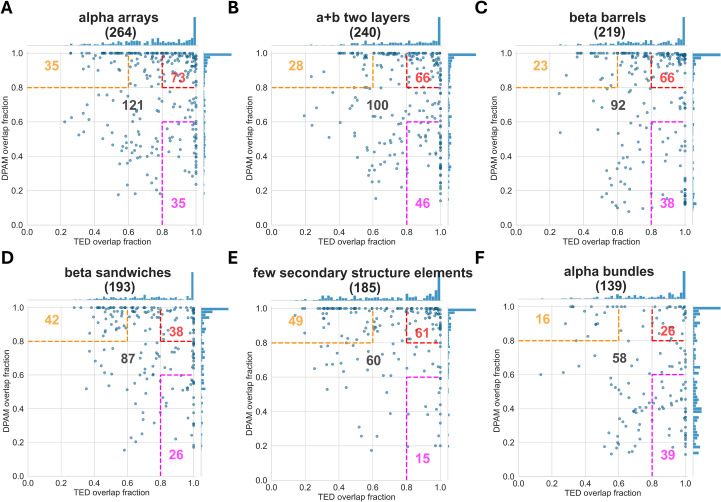
DPAM overlap fractions and TED overlap fractions for ECOD architectures that have a relatively large fraction of consistently assigned domains. The names of the architectures are shown as the titles of the plots with the number of domains in parentheses. Consistently defined domains are those with DPAM overlap fraction and TED overlap fraction greater than 0.8 (top right corner bounded by red dashed lines, with the number of domains shown in red). TED CNF domains largely embedded in DPAM domains have a DPAM overlap fraction less than 0.6 and a TED overlap fraction greater than 0.8 (bottom right corner bounded by magenta dashed lines, with the number of domains shown in magenta). DPAM domains largely embedded in TED CNF domains have TED overlap fraction less than 0.6 and DPAM overlap fraction greater than 0.8 (top left corner bounded by orange dashed lines, with the number of domains shown in orange). The numbers of domains not in the color-bounded regions are shown in black.

A total of 432 well-assigned DPAM domains have both TED overlap fraction and DPAM overlap fraction greater than 0.8 (top right corner of Fig 2 scatter plot), indicating that DPAM and TED CNF domain assignments are largely consistent. This agreement suggests that these domains are well-defined across both classification systems, likely representing functionally and evolutionarily stable protein families. Although this subset makes up only 17% of the whole set of well-assigned domains, it contains 17 unique ECOD architectural classifications and spans 117 distinct X-groups, demonstrating substantial structural and functional diversity. The ECOD architectures of “alpha-arrays”, “a+b two layers”, “beta barrels”, “beta-sandwiches”, and “few secondary structure elements” have a relatively large fraction of consistently assigned domains (Fig 5). The most common X-groups in this subset reveal a strong representation of beta-barrel and beta-sandwich architectures. Among the most frequent X-groups are SH3 domains (beta barrels), HTH (helix-turn-helix, alpha arrays), and Immunoglobulin-like beta-sandwich folds (beta sandwiches). These structures are commonly associated with protein-protein interactions, DNA-binding, and immune system functions. Other prevalent X-groups include Rubredoxin-like domains (few secondary structure elements), OB-fold (beta barrels), and Pili subunits (a + b two-layered structures). These domains often play critical roles in electron transport, nucleic acid binding, and bacterial adhesion, highlighting the diverse functional repertoire of this subset. The presence of RING/U-box-like and His-Me finger endonucleases further suggests that some of these domains are involved in ubiquitin signaling and DNA modification.

**Fig 6:**
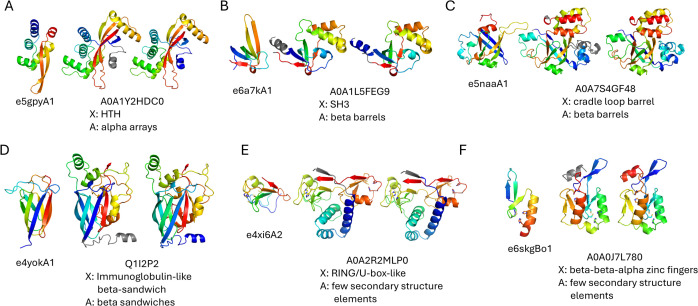
Examples of consistently assigned TED and DPAM domains. In each instance, the left structure is the ECOD domain template with the best DPAM probability score (with ECOD ID shown below), the middle structure shows DPAM domain-defined regions colored in rainbow; the right structure shows the TED CNF domain colored in rainbow. Regions not defined by DPAM or TED are colored gray. The UniProt accession number, the ECOD X-group and the ECOD architecture are shown for each domain.

Overall, this subset represents a well-defined, structurally diverse, and functionally versatile collection of protein domains. The consistent domain assignments between DPAM and TED indicate that these regions are evolutionarily stable, and their structural properties suggest that they participate in a wide range of biological processes. The relatively lower sequence similarity but strong structural conservation (X-group and architecture diversity) implies that many of these domains have undergone significant sequence divergence while maintaining their essential structural and functional roles. The structural variations of these domains are often reflected in the long insertions into the core structures (Fig 6A–6D). Manual inspection also revealed that in some consistently defined domains in the “few secondary structure elements” architecture, DPAM and TED-defined regions correspond to duplicated domains of two evolutionary units that are tightly packed against each other (Fig 6E: two RING finger domains and Fig 6F: two C2H2-type zinc finger domains).

### DPAM embedded in TED CNF domains

**Fig 7:**
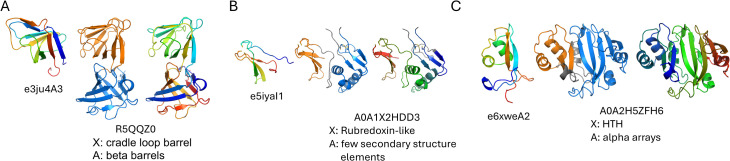
Examples of DPAM domains largely embedded in TED CNF domains. In each example, the left structure is the ECOD domain template with the best DPAM probability score, with ECOD id shown below the structure. The middle structure shows two DPAM domains colored orange and blue. The ECOD assignment is for the orange DPAM domain. For panels A and B, the blue DPAM domain has the same X-group assignment as the orange DPAM domain. For panel C, the blue DPAM domain is unassigned (DPAM confidence score less than 0.9). The right structure shows the TED CNF domain colored in rainbow. Regions not defined by DPAM or TED are colored gray. The UniProt accession number, the assigned ECOD X-group and the ECOD architecture are shown for each orange DPAM domain.

The scenario where a DPAM domain is shorter and embedded mainly in the corresponding TED CNF domain occurs in fewer cases: only 202 out of the 2490 well-assigned domains have a DPAM overlap fraction >0.8 and a TED overlap fraction <0.6 (top left corner of the Fig 2 scatter plot). This relationship suggests that in some cases, the DPAM domain definitions are more restrictive, possibly focusing on smaller, functionally distinct units within the broader TED-defined domain regions. On average in this subset, TED CNF domains span approximately 180 residues, while DPAM domains are only around 72 residues long, indicating that TED CNF domains may encompass multiple structural or evolutionary units beyond what DPAM classification captures. Indeed, manual inspection suggests that TED-defined regions in this subset often correspond to multiple structural units that DPAM successfully separates into distinct domains (Fig 7).

Despite the smaller number of cases, this group retains a notable degree of structural diversity, encompassing 10 unique ECOD architectural classifications and spanning 53 distinct X-groups. The most common X-groups in this subset include HTH (Helix-Turn-Helix) motifs, Rubredoxin-like folds, Immunoglobulin-like beta-sandwiches, and EGF-like domains, all of which are known for modular functions such as DNA binding, metal coordination, immune interactions, or extracellular signaling. Additionally, TED often clusters beta-beta-alpha zinc fingers and cradle-loop barrels, both of which serve important roles in protein-protein interactions and ligand recognition. The limited presence of secondary structure elements, combined with the relatively high number of distinct classifications, suggests that these DPAM-defined regions may represent highly specialized small domains. This aligns with the idea that these shorter domains could be functionally modular, acting as key interaction interfaces, catalytic sites, or binding elements embedded within a larger TED framework.

Functionally, this pattern suggests a form of functional refinement, where DPAM domains highlight the most critical, conserved portions of a broader TED CNF domain. These small, structurally focused regions may play essential roles in protein-protein interactions, signal transduction, or enzymatic activity. The presence of relatively few helices and strands implies that these domains might be more disordered or flexible, adapting their structures upon binding to specific partners. This contrasts with the previous subset, where larger DPAM domains scaffold smaller TED cores, indicating that domain organization and evolution can follow different strategies depending on functional demands. Further investigation into the functional roles of these domain segments could provide insights into their contributions to protein dynamics and molecular recognition.

## Conclusions

Our comparative analysis of TED- and DPAM-defined protein domains highlights both the challenges and opportunities in classifying the rapidly expanding landscape of predicted protein structures. By leveraging DPAM, which integrates AlphaFold-derived metrics, evolutionary information, and sensitive sequence/structure similarity searches, we demonstrated that a substantial portion of TED domains classified as candidate novel folds can be reinterpreted as highly divergent relatives of known structural families. This finding indicates that, while true structural novelties undoubtedly exist, many domains previously considered to be putative “new folds” may represent functional innovations arising from insertions, extensions, or recombination events within established folds.

The contrasting perspectives of domain definitions offered by TED and DPAM reflect broader methodological differences between classification frameworks such as CATH and ECOD. CATH emphasizes structural similarity and hierarchy derived from geometry-based clustering, which aligns closely with TED’s purely structural segmentation strategy. This approach excels at detecting recurrent structural motifs and cataloging topological diversity, but can risk over-segmentation, grouping repeats or insertions as independent domains without accounting for evolutionary continuity. In contrast, ECOD prioritizes evolutionary relationships inferred from sequence and structure evidence, a philosophy that parallels DPAM’s integration of evolutionary context. This approach unifies structurally diverse elements under common ancestry, but may sometimes obscure modular substructures that contribute to functional specialization. Thus, TED and CATH-style methods provide breadth in identifying structural diversity, while DPAM and ECOD-style approaches provide depth in tracing evolutionary connections.

The overlap patterns we observed underscore the complementary strengths and weaknesses of these approaches. TED frequently identifies embedded regions that DPAM unites into single evolutionary units, highlighting TED’s strength in structural modularity. Conversely, DPAM occasionally isolates smaller, conserved regions within broader TED domains, emphasizing its ability to refine classification by homology but at the potential cost of overlooking functionally distinct domains of insertions or repeats. Together, these findings reinforce that no single methodology fully captures the complexity of protein domain organization.

Importantly, one-third of DPAM domains overlapping with TED CNF domains that could be confidently linked to ECOD demonstrate the value of evolutionary context in annotating predicted structures. These assignments expand the reach of existing classification systems and reinforce the idea that distant homology can be detected even among proteins that diverge substantially in sequence and structure. At the same time, the large fraction of unassigned domains emphasizes the limits of current methods, pointing to regions of the protein universe where additional computational innovations or experimental validation will be required. Taken together, this study underscores the necessity of integrative strategies for accurate domain annotation. Structural segmentation (TED and CATH) and evolutionary classification (DPAM and ECOD) each contribute distinct but complementary insights. By reconciling structural novelty with evolutionary conservation, future efforts can achieve more accurate mapping of the protein universe, refine the definition of truly novel folds, and ultimately advance our understanding of protein evolution and function.

## Materials and methods

### DPAM domain parsing for proteins containing TED CNF domains

We used DPAM to parse domains from AlphaFold models of the 7232 proteins that contain the 7427 TED candidate novel-fold domains. The details of the DPAM algorithm are described elsewhere [26]. Briefly, using the predicted aligned errors (PAE) distributed with the AlphaFold predictions, regions that appear disordered or as linkers are excluded. DPAM predicts the probability for a pair of residues to belong to the same domain by their distance in the 3D structure, PAE between them, and whether this pair was aligned to the same ECOD domain based on sequence (HHsearch) and structure (DALI) searches. These probabilities were then used to cluster 5-residue segments into domains.

DPAM then utilizes a Neural Network [33] to classify the parsed domains in DPAM partitions and assign domains to ECOD homologous groups. We consider those globular domains with a DPAM confidence score above 0.9 to an ECOD entry and with a significant number of secondary structural elements (SSEs) as well-assigned domains. There are three categories of domains and regions that DPAM cannot confidently assign to an ECOD homologous group. 1) “Unassigned’‘ domains are globular domains with a significant number of SSEs that cannot be confidently assigned to a single homologous group (DPAM confidence score < 0.9). Often, these domains are sufficiently distant from known ECOD domains that additional expert considerations are needed, such as cofactor binding or functional inference from literature, to make an assignment. Frequently, these domains are candidates to expand the reference set by initiating a new homologous group, either with some partial homology signal to a known X-group (i.e., a new ECOD H-group within an existing X-group) or as an entirely new X-group. 2) “Simple topology” domains are composed of one or two SSEs and may contain intrinsically disordered or poorly predicted regions. Frequently, these domains have significant stability provided by non-SSEs (e.g., disulfide bonds, cofactors or metal binding sites). These regions may also be components of larger protein complexes and lack a globular structure outside this broader context. 3) “Partial” domains have confident homology to a much longer reference ECOD domain. These domains are usually the result of errors either in the query protein (i.e., genome annotation) or due to inconsistency within the reference set with respect to repeat and duplication (i.e., a query domain hitting a homologous domain duplication incorrectly annotated as a single domain).

The overlap fraction quantifies the agreement between domain boundaries defined by DPAM and TED. For any DPAM domain and a TED CNF domain from the same protein, we calculate two overlap fractions: the DPAM overlap fraction (number of intersection residues divided by DPAM domain length) and the TED overlap fraction (number of intersection residues divided by TED domain length). We focus our analysis on DPAM domains that show substantial agreement with TED CNF domains, requiring either overlap fraction to exceed 0.5.

### Manual analysis of protein domains

We manually analyzed a subset of DPAM well-assigned domains by examining sequence and structural similarity search results. To facilitate analysis, sequence conservation was calculated by AL2CO [34] and mapped to structures with the top conserved residues and disulfide-bond-forming cysteines highlighted in PyMOL. We inspected HHpred [27] results against PDB [35] and Pfam databases [7] and paid attention to conserved motifs among weak hits. Structural similarity searches were conducted by DaliLite [28], and for some proteins also by the Foldseek server [25]. Functional associations were analyzed by using the STRING web server [36].
